# Can Whole-Thyroid-Based CT Radiomics Model Achieve the Performance of Lesion-Based Model in Predicting the Thyroid Nodules Malignancy? — A Comparative Study

**DOI:** 10.1007/s10278-025-01584-6

**Published:** 2025-07-03

**Authors:** Wenxia Yuan, Jiayang Wu, Wenfeng Mai, Hengguo Li, Zhenyu Li

**Affiliations:** Medical Imaging Centre, First Affiliated Hospital of Jinan University, Tianhe District, No.613, Huangpu Avenue West, Guangzhou, 510632 Guangdong China

**Keywords:** Whole-thyroid, Machine learning, Computed Tomography (CT), Thyroid nodule, Thyroid carcinoma

## Abstract

Machine learning is now extensively implemented in medical imaging for preoperative risk stratification and post-therapeutic outcome assessment, enhancing clinical decision-making. Numerous studies have focused on predicting whether thyroid nodules are benign or malignant using a nodule-based approach, which is time-consuming, inefficient, and overlooks the impact of the peritumoral region. To evaluate the effectiveness of using the whole-thyroid as the region of interest in differentiating between benign and malignant thyroid nodules, exploring the potential application value of the entire thyroid. This study enrolled 1121 patients with thyroid nodules between February 2017 and May 2023. All participants underwent contrast-enhanced CT scans prior to surgical intervention. Radiomics features were extracted from arterial phase images, and feature dimensionality reduction was performed using the Least Absolute Shrinkage and Selection Operator (LASSO) algorithm. Four machine learning models were trained on the selected features within the training cohort and subsequently evaluated on the independent validation cohort. The diagnostic performance of whole-thyroid versus nodule-based radiomics models was compared through receiver operating characteristic (ROC) curve analysis and area under the curve (AUC) metrics. The nodule-based logistic regression model achieved an AUC of 0.81 in the validation set, with sensitivity, specificity, and accuracy of 78.6%, 69.4%, and 75.6%, respectively. The whole-thyroid-based random forest model attained an AUC of 0.80, with sensitivity, specificity, and accuracy of 90.0%, 51.9.%, and 80.1%, respectively. The AUC advantage ratios on the LR, DT, RF, and SVM models are approximately − 2.47%, 0.00%, − 4.76%, and − 4.94%, respectively. The Delong test showed no significant differences among the four machine learning models regarding the region of interest defined by either the thyroid primary lesion or the whole thyroid. There was no significant difference in distinguishing between benign and malignant thyroid nodules using either a nodule-based or whole-thyroid-based strategy for ROI outlining. We hypothesize that the whole-thyroid approach provides enhanced diagnostic capability for detecting papillary thyroid carcinomas (PTCs) with ill-defined margins.

## Introduction

Ultrasound (US) remains the primary imaging modality for thyroid nodule evaluation.[1] Its widespread adoption has significantly increased detection rates, contributing to the overtreatment [2]. Thyroid carcinoma is the most prevalent endocrine malignancy, with differentiated thyroid cancer—particularly in the case of papillary thyroid carcinoma (PTC)—showing favorable prognosis [3]. Notably, while thyroid nodules are detected in 65% of the general population, only 5–15% of these nodules are malignant [4]. Considering the high prevalence of thyroid nodules and the low incidence of thyroid carcinoma, The American College of Radiology Thyroid Imaging, Reporting and Data System (ACR TI-RADS) provides standardized risk stratification for thyroid nodules, its reliance on operator-dependent variability. the limited specificity of ACR TI-RADS (44.0%–67.3%) may prompt unnecessary biopsies or diagnostic thyroidectomies for clinically insignificant nodules [5]. Consequently, accurately identifying malignant lesions while minimizing invasive interventions is critical for optimal clinical management.[6]

Recent artificial intelligence (AI) advances have enabled radiomics to extract high-throughput features from conventional images, revealing tumor heterogeneity and malignancy-associated characteristics beyond human perception [7]. Radiomics-driven machine learning (ML) models using US or CT data show potential to surpass radiologists’ diagnostic accuracy in distinguishing benign from malignant nodules [8–10]. This method could reduces the influence of human factors. ML models that analyze the primary thyroid lesion as the region of interest (ROI) can achieve diagnostic accuracy comparable to or exceeding that of radiologists. However, this method remains resource-intensive, as current technologies face challenges in reliably identifying thyroid nodules with the same precision as lung nodules, despite progress in automated thyroid delineation. Current models exhibit suboptimal performance in thyroid nodule detection compared to their efficacy in pulmonary nodule identification. Furthermore, existing studies predominantly focus on isolated lesions, [7] which proves insufficient for nodules with unclear boundaries, multiple nodules, or micro-nodules(diameter ≤ 1 cm), where basic lesion segmentation fails to meet clinical needs. Because tumor biology encompasses both neoplastic cells and their associated stromal microenvironment, which dynamically influence cancer progression [11]. Manual image segmentation is also time-consuming, prone to inter-observer variability, and impractical for routine use.

To overcome these limitations, we propose a novel whole-thyroid segmentation strategy that achieves diagnostic performance comparable to individual nodule segmentation while enabling future full automation. This approach establishes a foundation for advancing automated thyroid segmentation and predicting lymph node metastasis in papillary thyroid carcinoma.

## Material and Methods

### Patient Selection

**Fig. 1:**
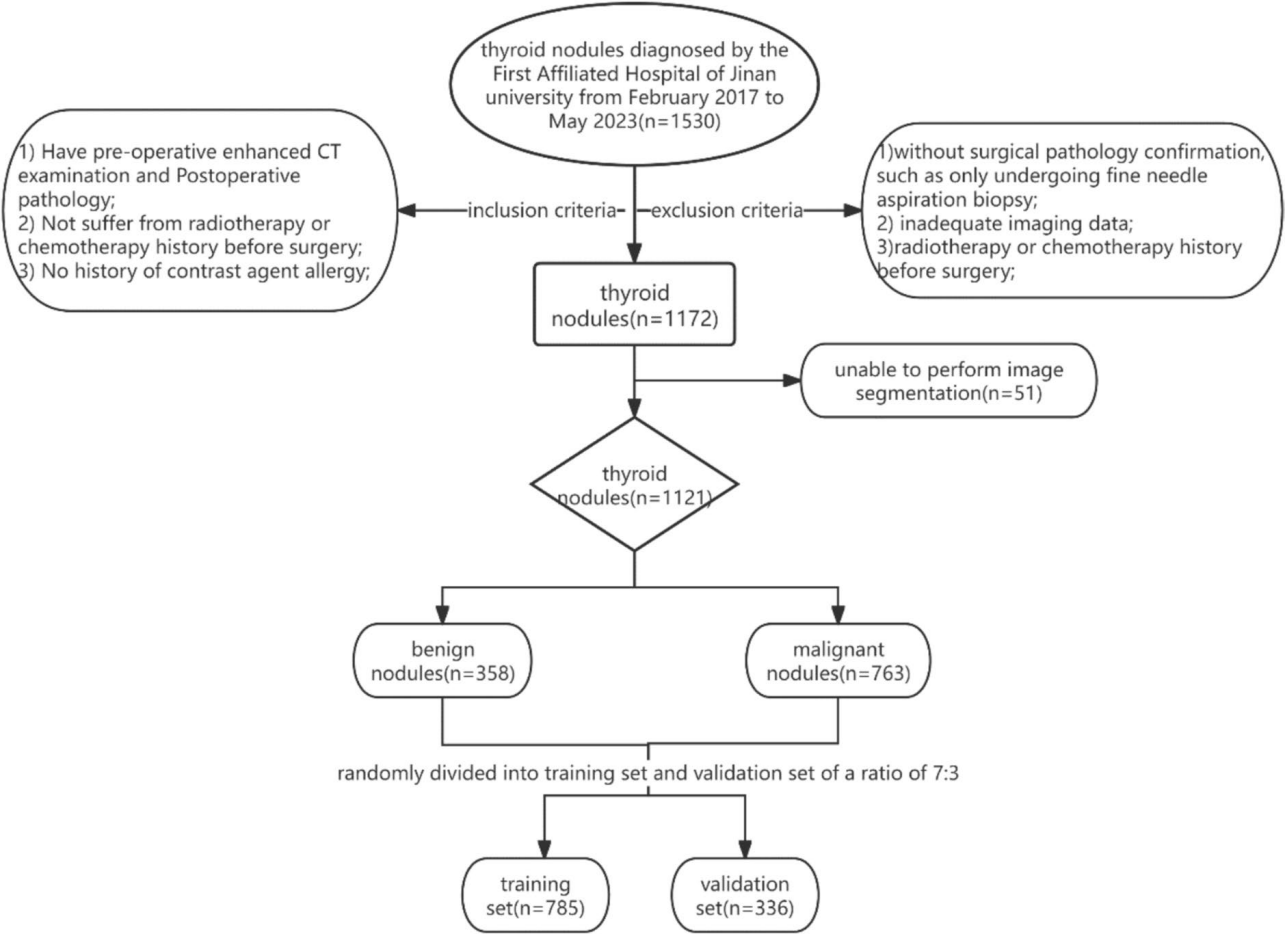
Standard flowchart for patient selection. *CT: *Computed tomography

This observational retrospective study received institutional review board approval from The First Affiliated Hospital of Jinan University (KY−2023–327). Informed consent was waived due to the retrospective design. From 1530 initially screened patients with thyroid nodules treated at The First Affiliated Hospital of Jinan University (February 2017–May 2023), 1121 met the following inclusion/exclusion criteria (Fig. 1):

Inclusion criteria:

- Histopathologically confirmed thyroid nodules with preoperative contrast-enhanced CT scans of diagnostic quality.
- No history of radiotherapy or other thyroid-directed therapies.
- No documented contrast media allergies.

Exclusion criteria:

- Incomplete clinical and image data.
- Absence of postoperative histopathological diagnosis (including fine-needle aspiration-only cases).
- Concurrent malignancies requiring neoadjuvant therapy.

Eligible patients were randomly allocated to training (*n* = 785: 534 malignant, 251 benign) and validation (*n* = 336: 229 malignant, 107 benign) cohorts at a 7:3 ratio. A total of 1130 whole-thyroid radiomic features and 963 nodule-specific features were extracted.

### Contrast Enhanced CT (CECT) Imaging Acquisition

All CECT images were digitally obtained from the Picture Archiving and Communication System (PACS) and associated databases using Toshiba 16-slice or 32-slice 64-layer spiral CT scanner, and GE CT (NeuViz Epoch Infinity 512-Layer Panoramic Multimodal CT). The acquisition parameters were: 300 mAs, 120 kV, a collimation width of 0.5 mm, a slice thickness of 3 mm, and a pitch of 0.8, the matrix size was 512 × 512 mm, and the field of view (FOV) measured 280 × 280 mm. Patients were positioned in a supine orientation, with the scanning range extending from the oropharynx to the superior margin of the clavicle. Following the initial scan, an enhanced scan was conducted using Iopamidol injection (Bracco, Italy), with a volume ranging from 50 to 100 ml, administered via a high-pressure syringe through the antecubital vein. Arterial phase and venous phase scans were performed at 30 s and 65 s post-injection, respectively. All patients provided informed consent prior to the examination, and an iodine allergy assessment was conducted before the procedure.

### Feature Extraction

**Fig. 2:**
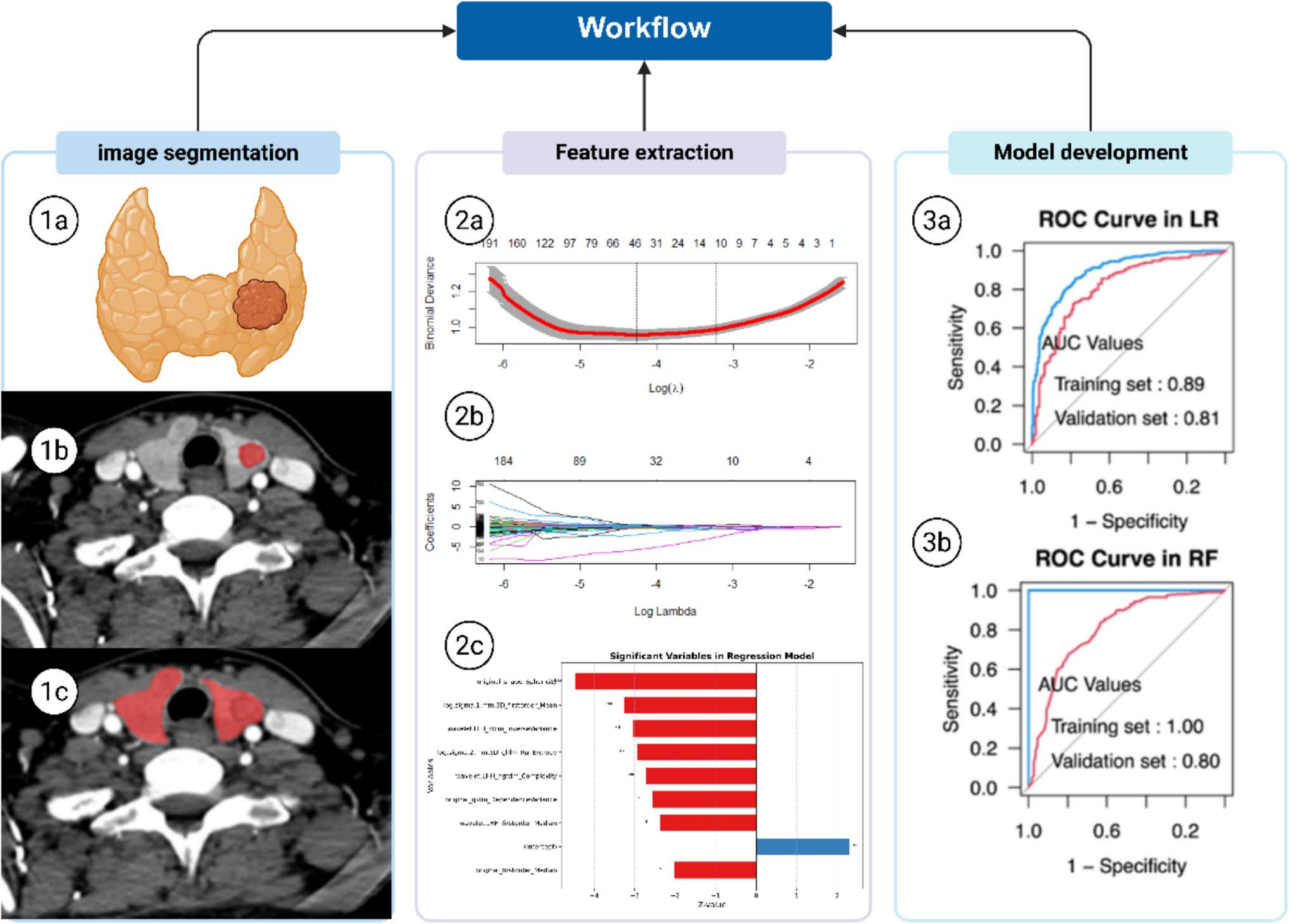
Image segmentation method. (1a) Simulated image of a left thyroid lobe nodule (1b) The thyroid nodule was outlining as ROI. (1c) the whole-thyroid was outlining as ROI. (2a-2c) Dimensionality reduction and feature selection of radiomic features using LASSO regression. (3a) LR model based on thyroid nodule achieved highest AUC in four nodule-based ML models. (3b) RF model based on whole thyroid achieved highest AUC in four whole-thyroid-based ML models. *ROI: *Region of Interest, US: ultrasound, CT: computed tomography, PTC: Thyroid papillary carcinoma, TI-RADS: Thyroid Imaging Reporting and Data System. ROC: receiver operating characteristic, AUC: area under curve, LR: Logistic Regression, RF: Random Forest, ML: machine learning

CT (CECT) images of the thyroid from all patients were acquired in DICOM format and converted to NRRD format using Python 3.9.12 (Python Software Foundation). For patients with multiple nodules, the nodule demonstrating the largest cross-sectional area and most clearly defined margins was selected. All thyroid nodules and whole-thyroid regions were delineated on arterial-phase images by two board-certified head and neck radiologists using ITK-SNAP. Each segmented nodule corresponded to its pathological size (Fig. 2 1b, 1c). Cases meeting any of the following exclusion criteria were flagged:

- Nodules unidentifiable on both non-contrast and enhanced CT despite pathological confirmation.
- Size discrepancy > 20% between imaging and pathological measurements. Intra-class correlation coefficients (ICCs) quantified segmentation reproducibility. To evaluate inter-observer agreement, two radiologists independently segmented 50 randomly selected nodules. Intra-observer consistency was assessed by having one radiologist re-segment the same nodules after a 2-week interval. Features with inter-/intra-observer κ > 0.75 were retained. The ‘pyradiomics’ package extracted 963 radiomic features from individual nodules and 1130 features from whole-thyroid regions.

### Feature Selection and Model Development

The imaging data of 1121 well-defined thyroid nodules were analyzed and randomly divided into training set (70%) and validation set (30%). Feature extraction and dimensionally reduction were applied to the training set using the Least Absolute Shrinkage and Selection Operator (LASSO) algorithm via the ‘glmnet’ package. (Fig. 2a, b) This approach enhanced model interpretability and predictive accuracy through feature selection, variable selection, and regularization, with feature selection being executed via 5-fold cross-validation. Four ML classifiers—Logistic Regression (LR), Decision Tree (DT), Random Forest (RF), and Support Vector Machine (SVM) were implemented using R’s ‘sklearn.model_selection’ and ‘e1071’ libraries. Each classifier was independently trained on two datasets: whole-thyroid regions and individual nodules. Final model performance was validated on the independent cohort, comparing results across both segmentation strategies.

### Statistical Analysis

Statistical analyses were conducted using R software (version 3.6.0, https://www.r-project.org). The ROC cure was generated utilizing the ‘pROC’ library, and the model’s predictive performance was evaluated based on the ROC curve and the area under the curve (AUC). Delong test was conducted utilizing MedCalc Software (version 20.022 MedCalc Software Ltd, Ostend, Belgium; https://www.medcalc.org) to ascertain whether differences in predictive performance existed between the complete thyroid and thyroid nodules. All statistical tests were conducted as two-sided, and a *p*-value of less than 0.05 was considered statistically significant.

## Result

### Patient Characteristics and Nodule Pathological Result

This retrospective study included 358 patients with benign nodules (nodular goiter, NG), while 763 exhibited malignant nodules (papillary thyroid carcinoma, PTC), including 189 cases in males and 574 cases in females within the PTC.

### Feature Extraction and Selection

LASSO was utilized for feature dimensionality reduction. For whole-thyroid-based radiomic, 49 features were selected from a total of 1130 features. For lesion-based radiomic, 47 features were selected from a total of 963 features. After statistically analyses, 33 and 43 optimal features were selected from whole-thyroid-based features and lesion-based features, respectively.

### Diagnostic Efficacy of Thyroid Lesion in Four ML Models

**Fig. 3:**
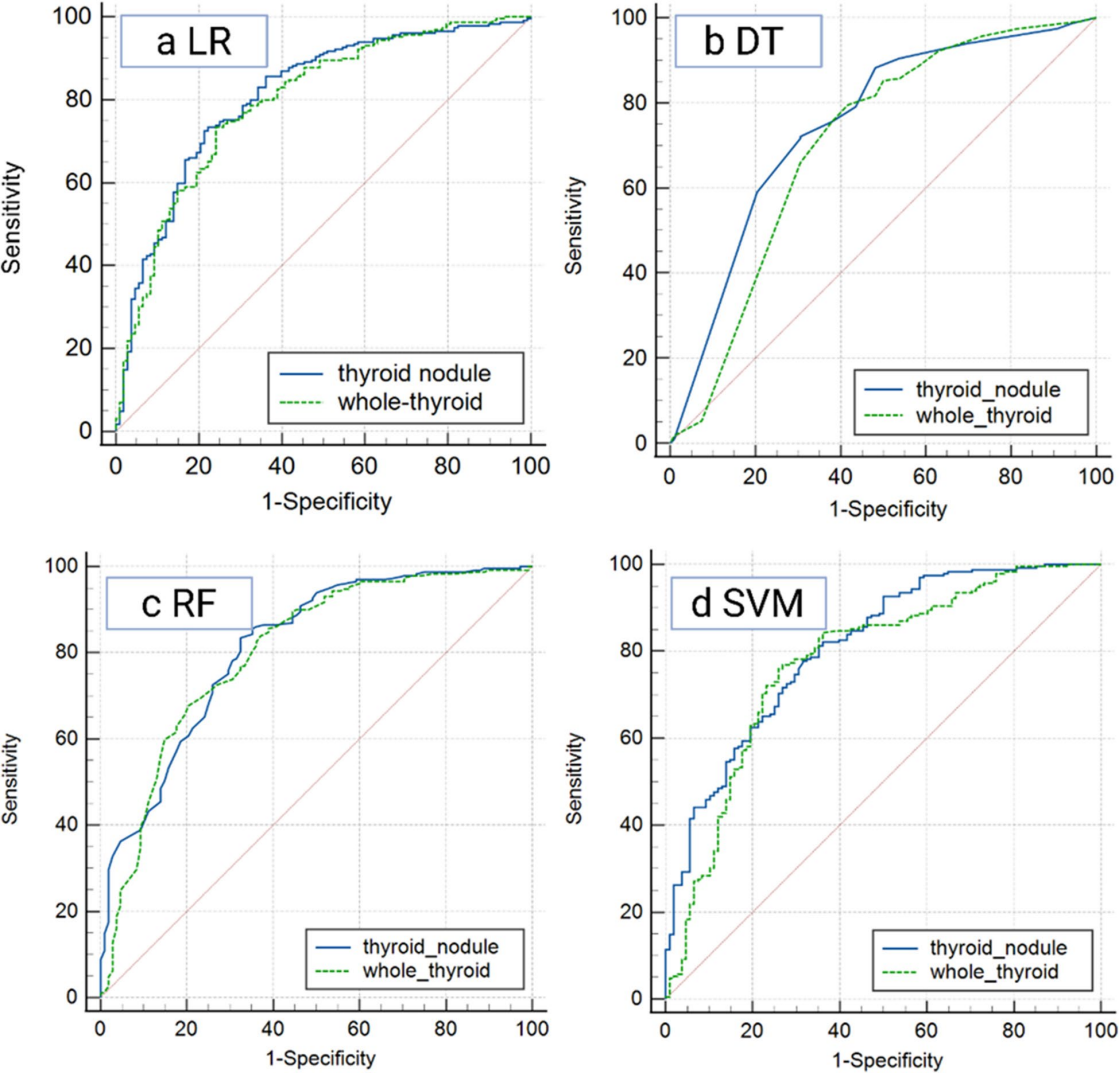
Comparison of the prediction performance of ML models for thyroid nodules and the whole-thyroid in the validation set. *LR: *Logistic Regression, DT: Decision Tree, RF: Random Forest, SVM: Support Vector Machine

We compared whole-thyroid-based and nodule-based model performance using LR, DT, RF, and SVM classifiers (Fig. 3). Whole-thyroid-based model achieved accuracy and AUC ranges of 0.75–0.80 (Table 1), with RF yielding the highest AUC (0.80) (Fig. 2 3b). For nodule-based models, accuracy and AUC spanned 0.75–0.81 (Table 2), because the RF model exhibits mild overfitting, LR yielding the highest AUC (0.81) (Fig. 2 3a). The advantage ratios of the whole-thyroid compared to the thyroid nodule segmentation method are shown in Table 3. Overall, the two segmentation methods differ in their focus: the thyroid nodule segmentation method performs better in predictive specificity, while the whole-thyroid model generally outperforms the nodule model in sensitivity and negative predictive value (NPV) (with an average increase of + 0.3% in sensitivity and an average decrease of − 1.4% in NPV for LR/DT/RF models). However, specificity and positive predictive value (PPV) remain challenging. For thyroid cancer screening, the whole-thyroid strategy with the LR model (sensitivity + 6.41%) is more suitable.

**Table 1:** Diagnostic efficacy of the whole-thyroid-based models in testing set

Model	LR	DT	RF	SVM
AUC	0.79 (0.73–0.84)	0.74(0.68–0.79)	0.80 (0.76–0.84)	0.77 (0.73–0.84)
Sensitivity	0.83	0.86	0.90	0.91
Specificity	0.59	0.46	0.54	0.29
PPV	0.81	0.77	0.81	0.73
NPV	0.62	0.60	0.72	0.60
Accuracy	0.75	0.73	0.78	0.71
F1	0.82	0.81	0.85	0.81

**Table 2:** Diagnostic efficacy of the nodule-based models in testing set

Model	LR	DT	RF	SVM
AUC	0.81 (0.76–0.86)	0.74 (0.69–0.80)	0.84 (0.80–0.89)	0.81 (0.76–0.86)
Sensitivity	0.78	0.90	0.94	0.92
Specificity	0.70	0.46	0.53	0.50
PPV	0.85	0.78	0.81	0.80
NPV	0.61	0.69	0.83	0.76
Accuracy	0.76	0.76	0.83	0.79
F1	0.81	0.84	0.87	0.85

**Table 3:** Superior percentage values of whole-thyroid compare to thyroid nodule

Model	LR	DT	RF	SVM
AUC	− 2.47%	0.00%	− 4.76%	− 4.94%
Sensitivity	+ 6.41%	− 4.44%	− 4.26%	− 1.09%
Specificity	− 15.71%	0.00%	+ 1.89%	− 42.00%
PPV	− 4.71%	− 1.28%	0.00%	− 8.75%
NPV	+ 1.64%	− 13.04%	− 13.25%	− 21.05%
Accuracy	− 1.32%	− 3.95%	− 6.02%	− 10.13%
F1	+ 1.23%	− 3.57%	− 2.30%	− 5.81%

### Evaluation of the Diagnostic Efficacy of Whole-Thyroid-Based Model in Ambiguous PTC

**Fig. 4:**
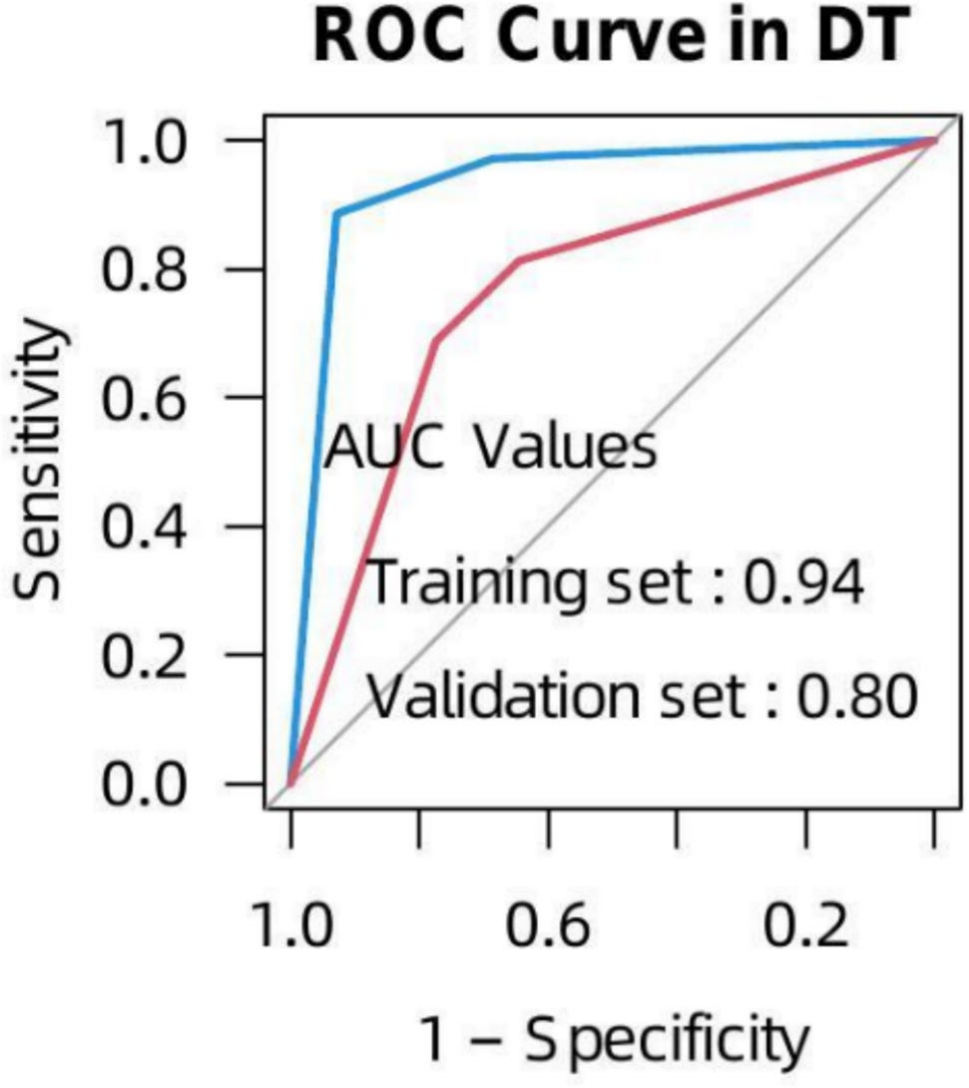
The predictive performance of the DT model constructed with the whole- thyroid-based ROI in 51 cases. *DT: *Decision Tree,

To validate the whole-thyroid model’s utility for PTC with ill-defined margins, we conducted a case–control analysis pairing 51 ambiguous PTC with 102 benign controls (1:2 ratio). The cohort was randomly divided into training (70%) and validation (30%) sets. In the validation set, DT achieved AUC 0.804 (95% CI: 0.684–0.925), sensitivity 81.3%, specificity 77.4%, and accuracy 78.7% (Fig. 4).

## Discussion

This study demonstrates that the whole-thyroid CT radiomics model achieves comparable diagnostic performance to nodule-based approaches in differentiating benign and malignant thyroid nodules. Notably, it exhibits enhanced capability in detecting ill-defined papillary carcinomas.

While radiomics has advanced thyroid nodule characterization using US and CT, reducing reliance on subjective radiologist interpretation[12, 13], current studies predominantly focused on nodule-specific analysis.[14] Few explore whole-thyroid imaging for malignancy prediction or lymph node metastasis assessment.[15, 16] Zhu et al. [17] demonstrated that peritumoral regions substantially contribute to predicting thyroid nodule malignancy, highlighting the significance of extranodular heterogeneity in radiomics analysis. Zhan et al. [18] distinguished thyroid follicular adenoma from adenocarcinoma using intranodular and perinodular CT radiomic features, further demonstrating the diagnostic value of extranodular regions in benign-malignant differentiation. Therefore, using thyroid nodules as the ROI presents critical limitations: (1) Nodules with poorly defined margins—particularly in thyroiditis cases, where reduced thyroid parenchymal density obscured lesion boundaries that pose challenges for accurate manual segmentation. (2) Given that thyroid nodules frequently present as multiple lesions, focusing on individual nodules fails to address clinical complexity in patients with multiple nodules.[19] (3) Manual segmentation of thyroid nodules is time-consuming and prone to inter-observer variability among clinicians. (4) Certain thyroid nodules are relatively small, making automation impractical. We propose adopting the whole-thyroid as the ROI for evaluating thyroid nodule malignancy. This approach replaces primary lesion segmentation, minimizing the subjectivity of manual delineation and can reflect overall extent of the thyroid gland.

The results of this study indicate that the nodule-based LR model achieves the best predictive performance. Originally proposed by Joseph Berkson, LR is widely used in medical research [20]. Peng et al. [21] compared three classification methods and found that SVM model performed best. For whole-thyroid-based ROI outlining strategy, Xu et al.[16] suggest that the LR model present the AUC was 94.2% in the test group. In contrast, our research not only found that the RF model achieves the best predictive performance, but also shown that using either thyroid nodules or the whole-thyroid as the ROI effectively predicts whether nodules are benign or malignant, with no significant difference in accuracy between the two strategies. This indicates that considering the whole-thyroid as the ROI is valuable, likely because it encompasses both nodular and surrounding features. This approach minimizes errors from manual delineation and enhances efficiency, and facilitating fully automated thyroid segmentation. Such progress paves the way for more practical software applications, setting the stage for further exploration in our subsequent analysis.

In 51 ambiguous boundary cases of PTC, lesions could not be segmented in either plain or arterial contrast-enhanced scans due to several reasons: (1) Nodules were too small or appeared in only one or two slices; (2) Clearly defined nodules were unidentifiable on CT but discernible on ultrasound; (3) Nodules visible on CT did not match the locations and sizes indicated by pathological evaluations. These nodules were confirmed by postoperative pathology as papillary thyroid microcarcinomas., highlighting a diagnostic oversight in imaging.

For 51 papillary thyroid carcinomas (PTCs) with ill-defined margins, we established a case–control cohort by matching with 102 benign nodules at a 1:2 ratio, dividing them into training and validation sets at a 7:3 ratio. The findings indicated that using the whole-thyroid as the ROI effectively predicts whether thyroid nodules are benign or malignant, enhances predictive performance for PTC with ill-defined margins, and reduces the false negative rate.

In subsequent research, we will prioritize the following objectives: Firstly, develop a DICOM-compliant automated segmentation model to standardize thyroid image analysis. Secondly, future multicenter studies with expanded cohorts should rigorously validate the diagnostic superiority of whole-thyroid radiomics for ill-defined nodules, particularly in cases with indeterminate features. Then, Investigate the prognostic potential of whole-thyroid radiomics features in predicting distant and lymph node metastases, with current focus on model optimization. Finally, establish a multimodal diagnostic framework integrating CT, ultrasound, and nuclear medicine data for comprehensive thyroid nodule management.

Our study has several limitations. Firstly, it is a single-center investigation, multi-center investigation should be conducted further to validate the generalizability and robustness of whole-thyroid-based model. Secondly, the enhanced imaging we used was limited to arterial phase images, plain scan and venous phase of the CECT were not explored, which we have planned to investigate in future research. Lastly, this study did not explore cervical lymph node metastasis, a critical factor influencing surgical decisions in thyroid cancer. We are currently conducting research to examine the predictive potential of whole-thyroid-based outlining strategy for identifying cervical lymph node metastasis.

## Conclusion

The whole-thyroid segmentation strategy demonstrates diagnostic efficacy comparable to nodule-based approaches. While whole-thyroid-based models do not demonstrate overall superiority over nodule-based approaches, they serve as a clinically viable alternative for cases with ill-defined lesion boundaries where reliable segmentation is unattainable. These findings validate the clinical value of comprehensive imaging analysis in improving diagnostic precision and provide a foundation for developing AI-driven automated segmentation pipelines.

## Data Availability

The data that support the findings of this study are included in the article; further inquiries can be directed to the corresponding author.
